# Finite Element Analysis and In-Situ Measurement of Out-of-Plane Distortion in Thin Plate TIG Welding

**DOI:** 10.3390/ma12010141

**Published:** 2019-01-03

**Authors:** Hui Huang, Xianqing Yin, Zhili Feng, Ninshu Ma

**Affiliations:** 1 Oak Ridge National Laboratory, 1 Bethel Valley Road, Oak Ridge, TN 37831, USA; fengz@ornl.gov; 2 Joining and Welding Research Institute, Osaka University, 11-1 Mihogaoka, Ibaraki, Osaka 567-0047, Japan; ma.ninshu@jwri.osaka-u.ac.jp; 3 State Key Laboratory for Mechanical Behavior of Materials, Xi’an Jiaotong University, Xi’an 710049, China; xqyin@xjtu.edu.cn

**Keywords:** welding distortion, finite element method, in-situ measurement, digital image correlation

## Abstract

Transient distortion of thin plate in the welding process usually has a complicated mode and large magnitude. Quantitative measurement and prediction of full-field distortion are challenging and rarely reported up to now. In this study, the out-of-plane distortion of a thin plate during the Tungsten Inert Gas (TIG) welding process was measured using the digital image correlation (DIC) method. A simulation model based on thermal elastic–plastic finite element method (FEM) and DIC measured geometric imperfection were developed for accurate prediction of the transient welding distortion. The numerical results and experimental data agreed very well in both out-of-plane deformation modes and magnitudes of the plate at different stages of welding. The maximum out-of-plane distortion was larger than 4 mm during welding which can cause instability of arc length and heat input. The distance change between welding torch and plate surface was investigated under different initial deflections of the plate before welding. The plate with flat geometry shows the minimum transient and final gap change. In addition, the relationship between heat input and welding distortion was clarified through a series of numerical analyses. Optimization of welding heat input can be performed based on numerical results to avoid excessive welding distortion.

## 1. Introduction

Fusion welding is widely used in the manufacturing industry, such as automobile and shipbuilding, to join metallic parts. Due to non-uniform heating and cooling, welding distortion and residual stresses are produced in the assembled structures. Compared with thick-section welding joints, thin-walled structures have more complex welding distortion which greatly affects the appearance and performance of welded products. Different from typical welding deformations, such as in-plane shrinkages and angular distortion, welding-induced out-of-plane distortion, especially buckling distortion, has more uncertainties in occurrence time, deformation mode, and magnitude because there are many influential factors, such as plate flatness, initial stresses, constraints, and heating conditions. In the design of light weighted structures, appropriate welding conditions and joint design should be considered to avoid excessive distortion during welding. Therefore, numerical simulation and coupon-level experiment need to be performed before actual welding.

There has been significant experimental work on welding distortion in thin plates. Terasaki et al. [1] carried out intensive experiments and simulations on the buckling of plates in different dimensions. New parameters were proposed to clarify the critical buckling condition. Tsai et al. [2] investigated the bifurcation and instability problem during the welding process by using an integrated experimental and numerical method. Transient stress, strain, and displacement were monitored in the numerical model to characterize the onset and halt of buckling. Pircher et al. [3] conducted the experiment on imperfections of tube plates after welding. Multiple waves of deflection were observed, and they were introduced in the numerical models for consequent buckling strength and ultimate strength analysis. Chu and Cho [4] measured the plate shape after welding in bead-on-plate and fillet welding joints. The welding speed, welding sequence, and constraint type were found to be influential factors of the buckling mode and magnitude. Most experimental studies were conducted to monitor the welding distortion at limited points in welding history or final distortion shape. The in-situ distortion field measurement is only presented by Shibahara et al. [5] and Guo et al. [6] so far.

The simulation of welding deformation and residual stresses has been increasingly used in researches and engineering as reviewed by Goldak and Akhlaghi [7]. It is an effective way to predict the welding distortion with lower cost compared with the experiment. Since the early 1990s, the inherent strain method based finite element method (FEM) has been applied in the prediction of buckling modes and magnitude after welding as noted by Ueda et al. [8]. By evaluating the residual plastic strain, it becomes much easier to conduct a simulation on a shell model by elastic FEM. Zhong et al. [9] investigated the buckling behavior of thin plate by using the inherent strain as an equivalent load. Various factors, such as plate thickness and the ratio of breadth to the length, were discussed. Michaleris and Debiccari [10] proposed a two-step method for simulation of buckling distortion in a welded structure in which a plane strain model was built to evaluate the residual stress on the cross-section perpendicular to the weld and then the stresses were applied into a three-dimensional model to perform elastic eigenvalue analysis. Nowadays, 3D thermal-elastic–plastic finite element (FE) analysis has become prominent for welding distortion and residual stress prediction [11,12,13,14] owing to its higher accuracy than simplified models. Nishikawa et al. [15] developed a fast iterative substructure method (ISM) in which the global FE model was divided into large linear zones and small nonlinear zones according to the transient temperature distribution during welding. Murakawa et al. [16] enhanced the ISM considering the characteristics of the welding-induced inherent strain. Huang et al. [17] modified the ISM and implemented large deformation theory in the welding deformation computation for thin plate welded joints. On the other hand, Ma et al. [18,19] proposed an accelerated explicit FEM for analysis of transient welding deformation and residual stress in large construction structures and automotive parts. Rong et al. [20] proposed a shell and 3D thermal mechanical model for effective prediction of laser welding-induced residual stress and distortion in 316L T-joint. Deng and Murakawa [21] combined the thermo-elastic–plastic FEM and the large deformation elastic FEM to predict the welding distortion of practical structures. The inherent strain theory and interface element were employed in the elastic FEM. Heinze et al. [22] analyzed the welding distortion of 5-mm thick plate with consideration of phase transformation. Through comparisons with experimental results, it was found that the distortion is sensitive to fractions of martensite, bainite, and ferrite. Wang et al. [23] studied the welding induced buckling in carbon thin plate by thermal-elastic–plastic FEM and elastic analysis based on inherent deformation respectively. Inherent bending deformation and initial deflection were found to be influential factors to trigger buckling.

To minimize the welding distortion, it is necessary to take advantage of both the numerical method and the experimental measurement. To simulate the welding-induced buckling distortion, the modelling procedure was presented by Yang et al. [24], the material model was discussed by Bhatti et al. [25], and the effect of initial deflection of thin plates was investigated by Huang et al. [26]. Yang and Dong [27] showed a computational framework for mitigation of buckling distortion. Deo and Michaleris [28] validated the numerical simulation models and then replaced expensive experimental trials for prevention of buckling distortion. Because the quantitative numerical simulation of transient distortion in welding needs to be validated by the in-situ measured information, the development of advanced measuring techniques for the full-field welding deformation is especially important. In the present study, the out-of-plane distortion of a Tungsten Inert Gas (TIG) welded bead-on-plate was investigated by both in-situ digital image correlation (DIC) measurement and 3D thermo-mechanical analysis. The transient welding distortion of a rectangular plate of Q235 steel was measured during the welding and cooling processes. The initial geometry measured in the experiment was mapped into the numerical model so that the deformation mode and magnitude in the time history can be accurately simulated. To clarify possible ways to minimize the out-of-plane distortion in welding, parametric studies on heat input and initial deformation were performed using thermal elastic–plastic FEM solver JWRIAN developed by authors. The DIC full view of initial plate geometry and high-fidelity computation offers an accurate way of transient welding distortion prediction and control.

## 2. In-Situ Measurement of Welding Distortion

### 2.1. Welding Experiment and Measuring System

**Figure 1:**
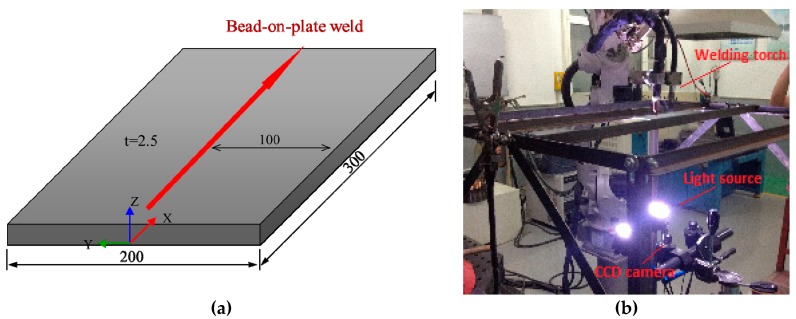
Welding and measuring setup. (**a**) Dimension of plate (**b**) Measuring system.

A rectangular plate of hot rolled mild steel Q235 was prepared for the welding experiment as shown in Figure 1a. The dimension of the plate was 300 mm × 200 mm × 2.5 mm. It should be noted that, initial residual stresses in steel plates can be large depending on the plate thickness and heat treatment condition [8,29]. Before welding, the plate was heat treated in a furnace by raising the temperature to 600 °C with a heating rate of 150 °C/h. The holding time at peak temperature was 2 h. The plate was then gradually cooled to 300 °C before being taken out for natural cooling. Through such heat treatment, initial residual stresses could be greatly released so that its influence on welding distortion were limited. Figure 1b shows the experiment setup. Tungsten Inert Gas (TIG) welding process was employed to avoid the effect of filler metal and reinforcement. Therefore, the material and geometrical model could be kept simple for mechanical analysis by the finite element method. The welding piece was placed on the multi-functional welding platform without restraint so it could deform freely. The welding parameters are summarized in Table 1. After welding, the specimen cooled naturally in air with ambient temperature about 20 °C. No additional heat treatment was performed after cooling.

### 2.2. Welding Distortion Measurement by DIC Technique

**Figure 2:**
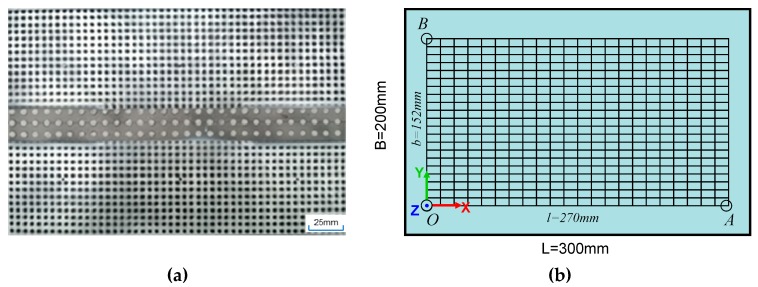
Distortion measurement by the digital image correlation (DIC) technique. (**a**) speckle paint on plate back side, (**b**) point grid for data extraction.

The digital image correlation method was proposed by Chu et al. [30] for measuring deformation and strain. Now it is widely used in experimental test as the non-contact measuring method [31,32]. Using the DIC technique to measure deformation in welding has two challenges: the intensive arc light and filler metal dust can affect measuring accuracy. Recently, researchers Xiao et al. [33] and De Strycker et al. [34] successfully extended the DIC technique to measure welding distortion. Chen et al. [35,36] measured strains in welded plates and compared the results with the numerical model. The digital image correlation system XJTU-DIC [6] for 3D full-field deformation and strain measurement was employed in this research. In the experimental measurement, the speckles were prepared on the back side of the plate before welding. The DIC camera was placed underneath the weld plate with a distance of about 800 mm. Before speckle painting, the region near the weld was sandblasted to create enough roughness and reduce light reflection. Then we prepared the speckle pattern using an inorganic adhesives Cerastil C-3 which has a high-temperature resistance (up to 1460 °C) and has a thermal expansion coefficient (12.9 × 10^−6^/°C) very close to steel. Thus, the adhesive can deform with the plate surface to the same extent during the welding without introducing error by thermal expansion mismatch. The dimension of the measuring zone was selected as 270 × 152 mm as observed in Figure 2. The measured accuracy on *x*, *y* and *z* coordinates was 0.01 mm. The coordinates of the points were measured at a time interval of 0.5 s. The sampled data was output on a grid of 38 columns and 22 rows, which were used for comparison with the analyzed results of FEM. Coordinates of all nodal points were transformed by fixing the out-of-plane displacement Uz at points O, A, and B. Meanwhile, the in-plane displacement Ux at point O, B, and Uy at O were also fixed. The numerical model was also constrained in the same way for a direct comparison of welding distortion.

**Figure 3:**
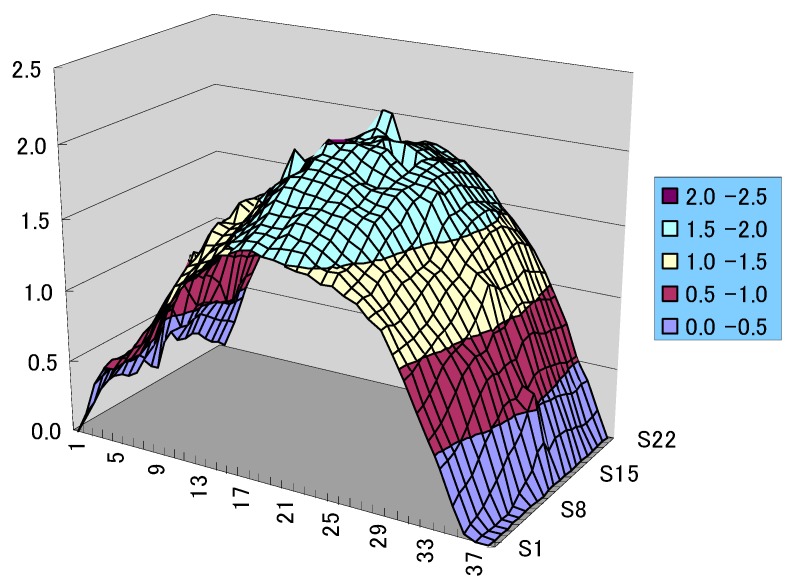
Out-of-plane geometrical imperfection scanned by DIC (Unit: mm).

We know that the distortion of a structure under external load is sensitive to initial stress and geometrical imperfection. Therefore, the initial deformation of the plate before welding was captured by DIC measuring technique. As shown in Figure 3, the original plate has a sinusoidal shape with a magnitude of about 2 mm which is 80% of plate thickness. Here, 1~37 in horizontal axis denote dataset number in *x*-direction, and S1~S22 denotes dataset number in y-direction. In the numerical model, initially flat geometry was modified by mapping the initial deformation from the measured points to FE nodes. Then thermo-mechanical analysis was performed based on the measured geometry, and the welding-induced distortion was evaluated by comparing the current coordinate with initial coordinates.

## 3. Finite Element Analysis

The welding process involves thermal–mechanical–metallurgical behavior which makes a coupled simulation very challenging. From the viewpoint of the heat transfer process, the heat converted from the mechanical process is much smaller than the welding heat. Though research by Sun et al. [37] and Coret et al. [38] indicates external load can induce phase transformation and dynamic ferrite recrystallization in low carbon steels, the strain level in the case of welding is much lower than those of their experiment. In addition, the microstructural change in SS400 steel has a minor effect on stress and strain as demonstrated by Nagai et al. [39]. Thus, it is reasonable to perform a thermal–mechanical analysis in the sequentially manner.

### 3.1. Transient Thermal Elastic–Plastic FEM

**Figure 4:**
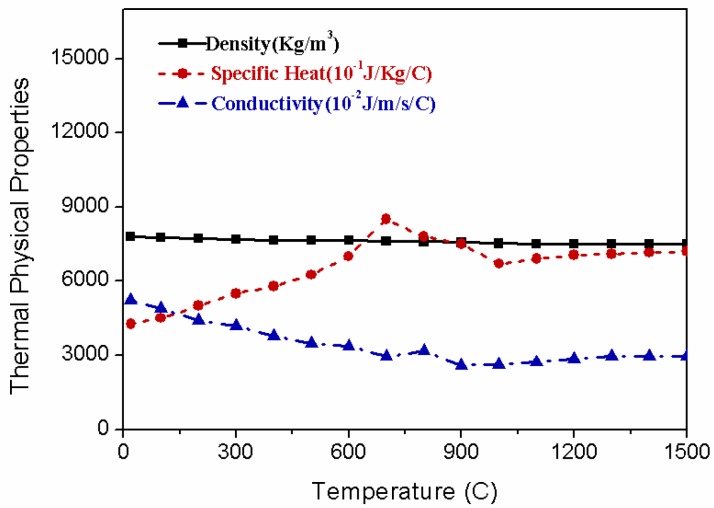
Thermal physical properties of Q235 as a function of temperature.

The transient thermal elastic–plastic FEM was employed to analyze the TIG welding process of thin plate. The equation of thermal conduction [8] with a volumetric heat generating rate *q* for TIG welding can be described by Equation (1).

(1) $$\rho c\frac{\partial T}{\partial t}-\nabla \cdot \left(k\nabla T\right)-q=0$$

where, ρ is the material density, *c* is the specific heat, and *k* is the heat conductivity. These material properties are temperature dependent as shown in Figure 4.

The distribution of volumetric heat in a moving ellipsoidal zone can be given by the following equation in the numerical model.

(2) $$q=\frac{6\sqrt{3}Q}{abc\pi \sqrt{\pi }}\begin{array}{c} e^{-3{\left(x-x_{0}\right)}^{2}/a^{2}}e^{-3{\left(y-y_{0}\right)}^{2}/b^{2}}e^{-3{\left(z-z_{0}\right)}^{2}/c^{2}} \end{array}$$

where *Q* denotes the heating power which can be calculated from the welding current and voltage *Q* = *ηUI*. The heat efficiency *η* was taken as 0.8. *x*, *y*, *z* are coordinates for a point where the volumetric heat is applied. *x*_0_, *y*_0_, *z*_0_ are coordinates of the heat source center at the current time. The parameters *a*, *b*, *c* denote the semi-axes of the ellipsoidal zone in welding direction, a transverse direction, and plate thickness direction, respectively.

On the free surface of plates, the heat is transferred to the air and surroundings through convection and radiation. Radiation can be taken into account by convection using an equivalent heat transfer coefficient as described by Ueda et al. [8]. The equivalent heat flux going out of the surface can be written as:

(3) $$q_{S}=\beta \left(T-T_{a}\right)$$

The ambient temperature *T_a_* and initial temperature of the steel plate were both 20 °C. *β* is the equivalent coefficient of the heat transfer. The energy equation is integrated over the finite element domain and solved in time increment form.

In thin-walled structures, the out-of-plane deformation is greatly affected by the in-plane deformation components. The temperature gradient through the thickness is also critical to the formation of bending stress and strain. For the thermal mechanical problem, the total strain consists of three basic components if phase transformation induced strain in neglected.

(4) $$\varepsilon_{ij}^{total}=\varepsilon_{ij}^{elastic}+\varepsilon_{ij}^{plastic}+\varepsilon_{ij}^{thermal}$$

where *ε_ij_^elastic^*, *ε_ij_^plastic^*, *ε_ij_^thermal^* are the elastic strain, plastic strain and thermal strain, respectively. Subscripts *i*, *j* take the value of 1~3 independently.

To accurately predict the deformation in thin plates, 3D thermal-elastic–plastic FEM together with large deformation theory is necessary. The Green strain expression in the Lagrangian form can be used as described by Zienkiewicz and Taylor [40].

(5) $$\varepsilon_{ij}^{total}=\frac{1}{2}\left(\frac{\partial u_{i}}{\partial X_{j}}+\frac{\partial u_{j}}{\partial X_{i}}+\frac{\partial u_{k}}{\partial X_{i}}\frac{\partial u_{k}}{\partial X_{j}}\right)$$

where, *X_i_* is the initial coordinate component of a material point, and *u_i_* is the displacement component.

The thermal strain component can be expressed as below:

(6) $$\varepsilon_{ij}^{thermal}=\alpha \left(T-T_{REF}\right)\delta_{ij}$$

where *α* is coefficient of thermal expansion (CTE), *T* is the current temperature in the element and *T_REF_* is reference temperature. *δ_ij_* is Kronecker delta which equals to 1 if *i* equals to *j* and 0 for other cases.

**Figure 5:**
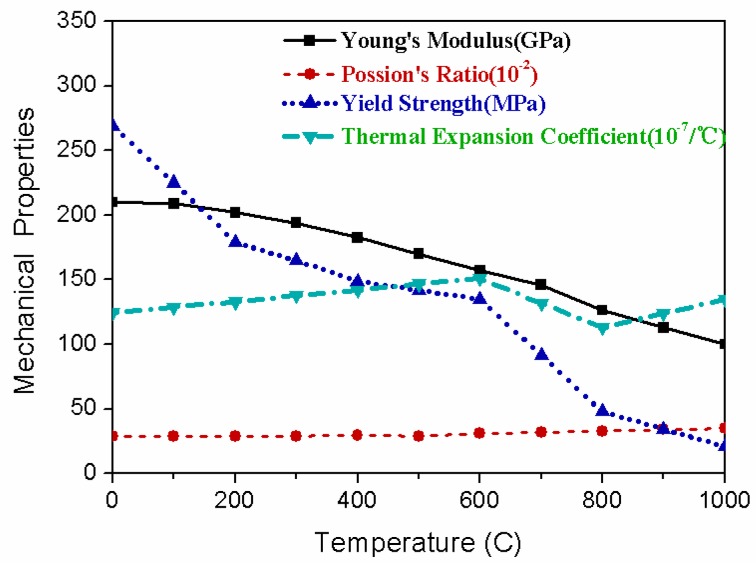
Mechanical properties of Q235 as a function of temperature.

The elastic strain has a linear relationship with stress components *σ_ij_* given by the following equation.

(7) $$\varepsilon_{ij}^{elastic}=\frac{1+\upsilon }{E}\sigma_{ij}-\frac{\upsilon }{E}\sigma_{kk}\delta_{ij}$$

where, *E* is Young’s modulus, and *υ* is Poisson’s ratio. The detailed mechanical properties including the yield strength and thermal expansion coefficient are plotted in Figure 5. The temperature dependent material properties of Q235 are referred to SS400 [41] since the two materials have very close chemical compositions.

Isotropic hardening at the plastic deformation zone was assumed, and the increment of plastic strain can be expressed as:

(8) $$d\varepsilon_{ij}^{plastic}=\frac{3}{2}\frac{d{\overset{⌢}{\varepsilon }}^{P}}{\overset{⌢}{\sigma }}s_{ij}$$

where, *S_ij_* is the deviatoric stress components, $\overset{⌢}{\sigma }$ and $d{\overset{⌢}{\varepsilon }}^{P}$ is effective stress and effective plastic strain respectively.

Given the displacement increment, the above equations can be used to obtain the updated stress field and nodal force vector. After solving the simultaneous linear equations, a new displacement field can be obtained. The iteration computations are performed until the resultant nodal force becomes very small compared with internal forces. The stiffness matrix is updated at the beginning of each time step to balance the solution of accuracy and efficiency.

### 3.2. Computation Flowchart

**Figure 6:**
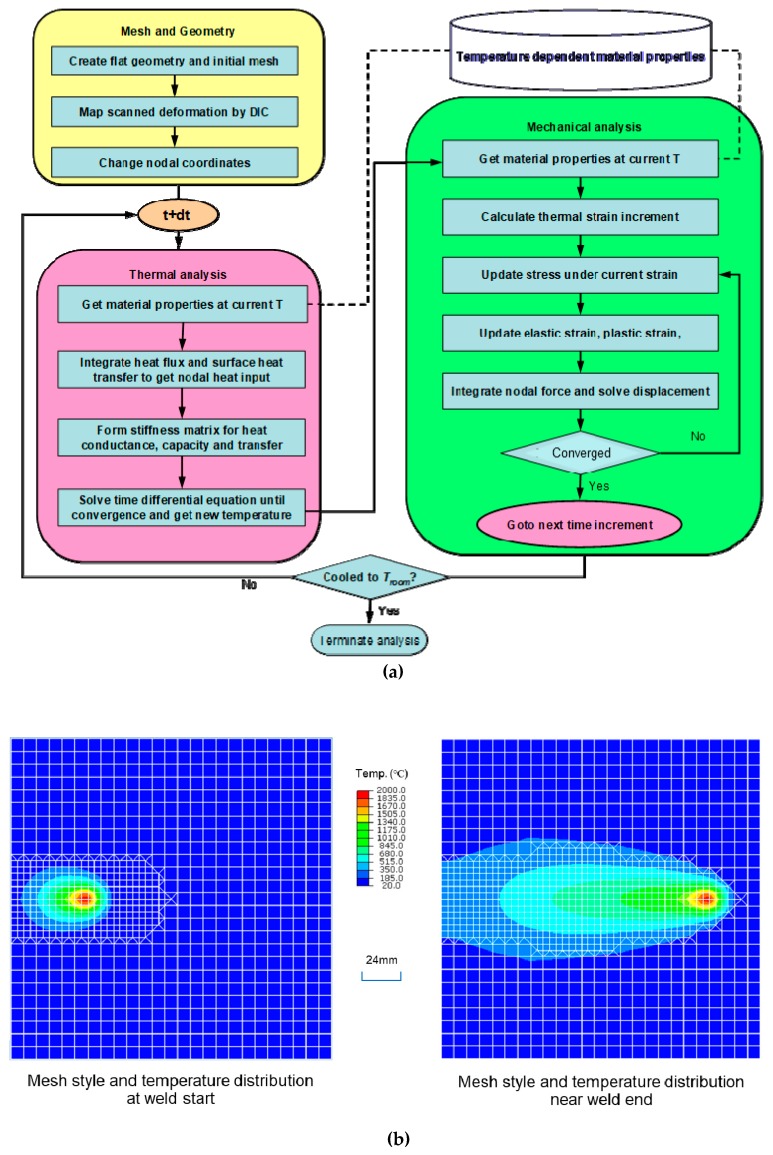
Analysis procedures and dynamic mesh refining scheme in research solver JWRIAN. (**a**) Computation flowchart; (**b**) Dynamic mesh refining scheme.

We employed an efficient solver JWRIAN (Joining and Welding Research Institute ANalysis code) to carry out the thermal elastic–plastic finite element analyses. The computational procedures are summarized in Figure 6a. In this study, the influence of the initial geometrical imperfection was considered. The flatness of the plate before welding was introduced into the finite element model by mapping the measured initial coordinates. Thermal analysis was firstly carried out, and the temperature data was written into a file. The mechanical analysis was then performed by reading temperature from the file at each time step. To save computation time, the dynamic mesh refining method developed by Huang and Murakawa [42,43] was adopted in the thermo-mechanical analysis. As shown in Figure 6b, the mesh around the heat source is automatically refined, and the region of mesh refinement is moving with the welding heat source. To predict the welding distortion accurately, it is necessary to consider the residual stress effect in the numerical model by advanced simulation and measurement. Residual stress can be obtained through coupled simulations from rolling, heat treating, stamping to welding. Due to the heat treatment employed in the study, residual stress before welding was not considered.

**Figure 7:**
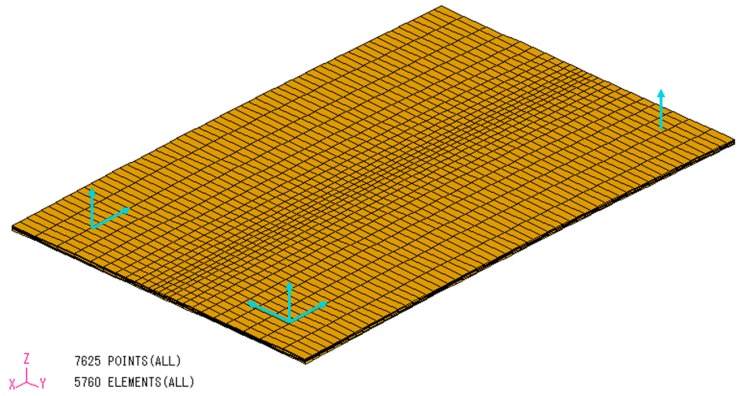
Finite element model of the plate by Tungsten Inert Gas (TIG) welding.

Figure 7 shows the finite element model of the experimental rectangular plate by TIG welding. The number of nodes and elements in the background mesh [42] are 7625 and 5760, respectively. The elements are 8-node hexahedra type. The boundary condition shown in the figure eliminates the rigid body motion only which is the same case in the experiment.

## 4. Results and Discussion

### 4.1. Comparison of Welding Distortion in Baseline Model

**Figure 8:**
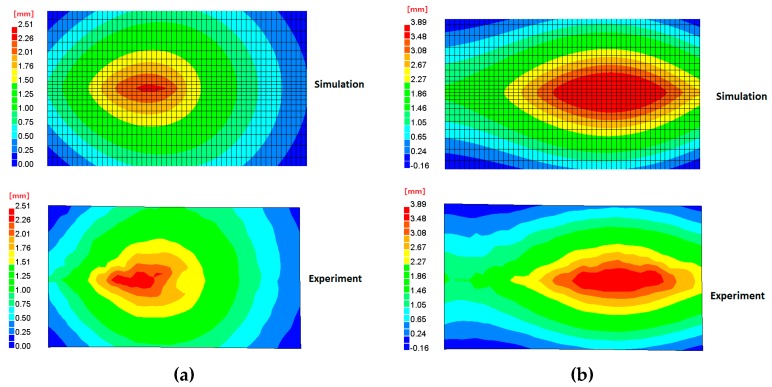

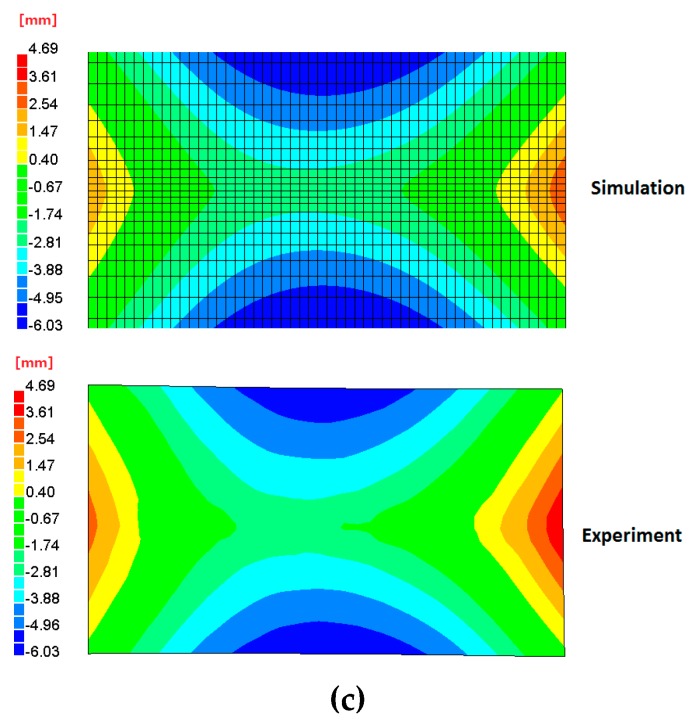
Comparison of plate out-of-plane deformation contour at different time. (**a**) *t* = 30 s; (**b**) *t* =60 s; (**c**) *t* = 400 s.

**Figure 9:**
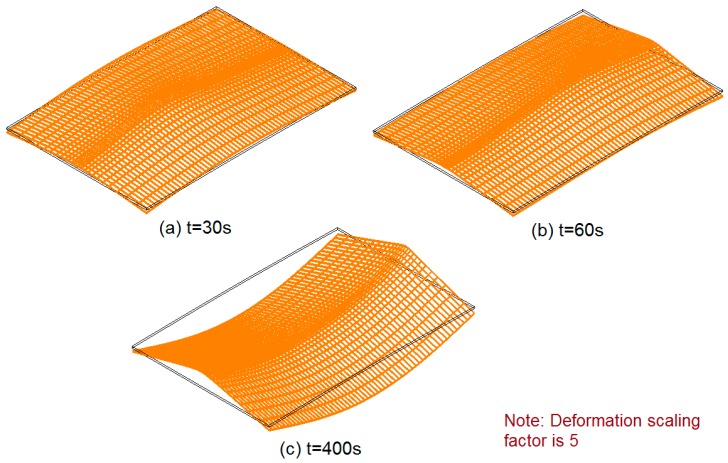
3D plots of distorted plate by simulation.

The transient out-of-plane deformations at *t* = 30 s, 60 s, 400 s were compared between the measurement and simulation. In Figure 8a, the deformation of the plate was like a dish shape when the welding torch moved to the middle of the specimen. The depth of the plate deformation was the order of the plate thickness. In Figure 8b, the deformation mode became a spindle shape when the welding has just finished at the end of the plate. At this moment, the region near the weld line was at the high temperature, so they behaved softer compared with the surrounding part. In Figure 8c, the deformation exhibited a saddle shape which had the magnitude of about 10 mm. The maximum temperature at *t* = 400s was about 60 °C which was very close to the room temperature. High tensile stress was generated in the vicinity of the weld, and the compressive stress was distributed over a large area of the plate as a result of load equilibrium. When the plate started to develop longitudinal bending, the transverse bending was also produced due to the Poisson’s effect. To better visualize the shape of the plate during welding, 3D plots of distorted finite element model are shown in Figure 9. The obtained results demonstrate that both the transient deformation mode and magnitude of the welded plate agreed very well between simulation and experiment. Specifically, it was found that the plate had a quite large deformation (>4 mm) when the welding torch was still on the plate. This phenomenon can become very severe in practical manufacturing because the welding process will be interrupted if the distance between the torch and the plate surface is too short. On the other hand, if the distance is too long, the quality of the weld is deteriorated due to variable heat input.

**Figure 10:**
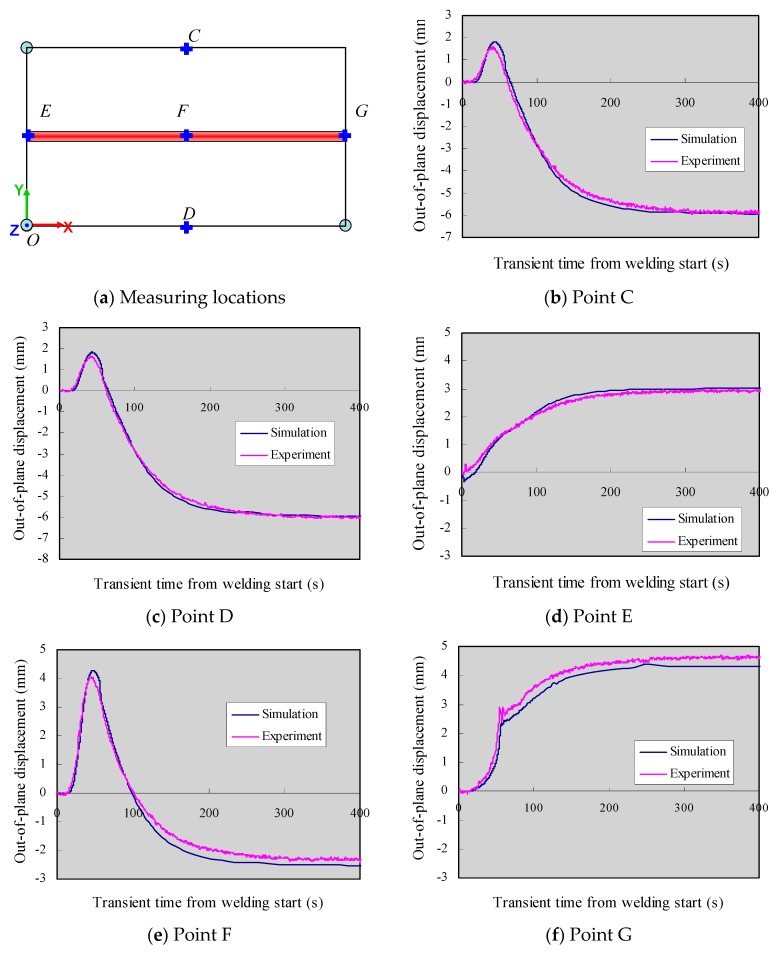
Out-of-plane displacement at various locations during welding process. (**a**) Measuring locations; (**b**) Point C; (**c**) Point D; (**d**) Point E; (**e**) Point F; (**f**) Point G.

To validate the simulation accuracy in the whole time history, the transient displacements were compared at several points C~G as depicted in Figure 10a. Clearly, the points C, D, and F moved upwards during welding, and they started to drop off at cooling. Point E gradually moved in a positive direction while point G showed two different stages in the displacement change history. This is caused by the welding torch leaving the plate end and subsequent drastic cooling. Comparing displacement at the points C and D, it can be confirmed that experimental out-of-plane distortion and its modes were well reproduced by simulation. In all measured locations, the displacements did not change much from t = 300 s, which indicates the stress distribution reached a steady state.

### 4.2. Effect of Initial Plate Shape on Distortion Mode

**Figure 11:**
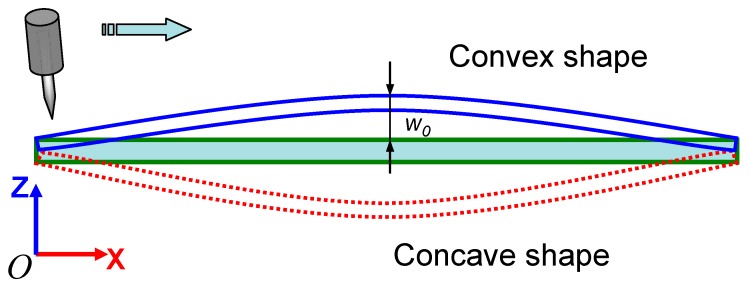
Different shapes of the plate before welding.

To clarify the influence of the initial geometric imperfection on the transient and final distortion mode, three cases with an idealized flat shape, concave, and convex initial shapes shown in Figure 11 were considered. The welding conditions and analysis procedures were the same as the previous experimental case. The distance change between the welding torch and the plate was traced dynamically to study its effect on welding process instability.

**Figure 12:**
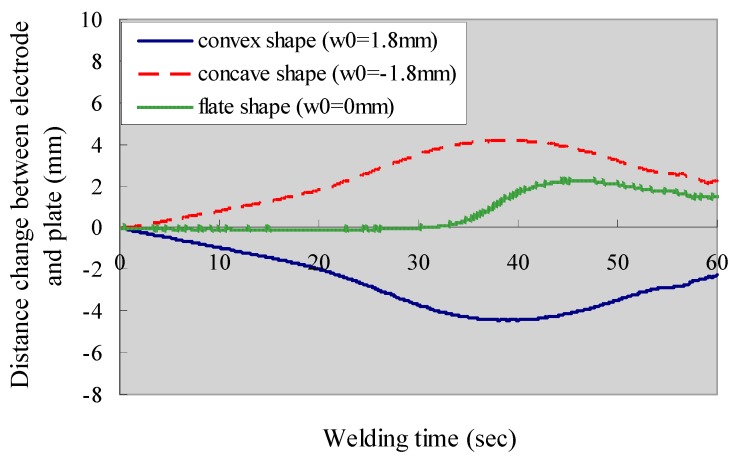
Effect of plate shape on the transient distance change between electrode and plate.

From Figure 12, it can be observed that the plate will become closer to the electrode when the initial shape of the plate is convex. The maximum change of distance will be 4 mm at about 40 s from the start of the welding. The welding heat input will be affected due to the change of arc length. Tashiro et al. [44] have shown that a decrease of arc length by 2 mm will induce a 12% decrease in welding power for a constant current (CC) power source. On the other hand, the plate tends to move away from the welding torch when the initial shape of the plate is concave. The magnitude of the distance change is slightly lower than the convex case. In either case, the large change of the distance between the welding torch and plate surface may induce the instability of welding arc and, thus, may lead to a poor welding quality. If the plate is flat without any initial deflection, the distance between torch and plate surface changes only 2 mm. Therefore, it is advisable to straighten the plate before welding or set appropriate fixtures during welding.

**Figure 13:**
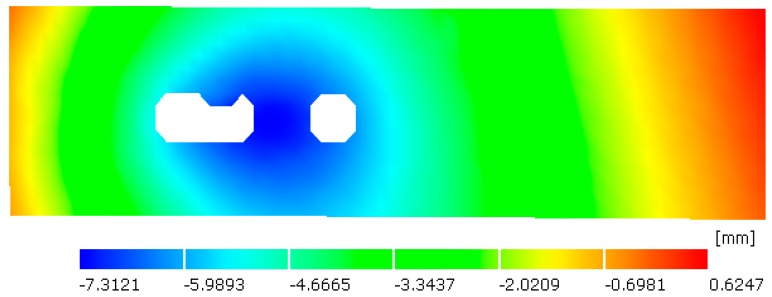
Welding distortion of a plate with arc blowout (DIC camera viewing from plate backside).

In a more severe case, the welding process can be interrupted if the excessive distortion has occurred during welding. This problem was observed in welding longer plates with a length of 600 mm. In several cases, arc blowout occurred in the early stage of welding due to the contact between the torch and plate surface. The distance change between the torch and plate surface was almost 7 mm at the time of arc blowout as shown in Figure 13. To ensure high-quality weldment, automatic adjustment of electrode height should be used in practical welding engineering.

### 4.3. Parametric Study on the Effect of Welding Heat Input

**Figure 14:**
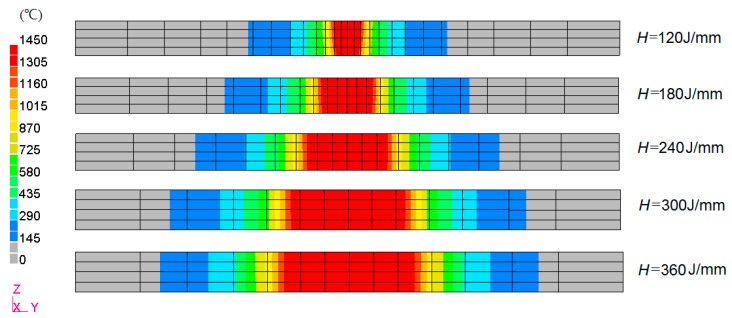
Weld penetration under various heat input condition.

The welding heat input has a large influence on welding distortion, such as the angular distortion and transverse shrinkage. In this study, the effect of heat input as denoted by *H* = *Q*/*v* on the out-of-plane distortion was investigated numerically through the thermal mechanical FE analysis. To ensure the appropriate weld penetration in each case, the mesh size was adjusted with the heat input proportionally. Figure 14 shows the mesh at the cross section and calculated penetration shape under the different welding heat input. For a welding energy of *H* = 120 J/mm, the corresponding width of the weld pool was less than the plate thickness which was 2.5 mm. In the experimental case with the heat input *H* = 360 J/mm, the penetration width was nearly 8 mm.

**Figure 15:**
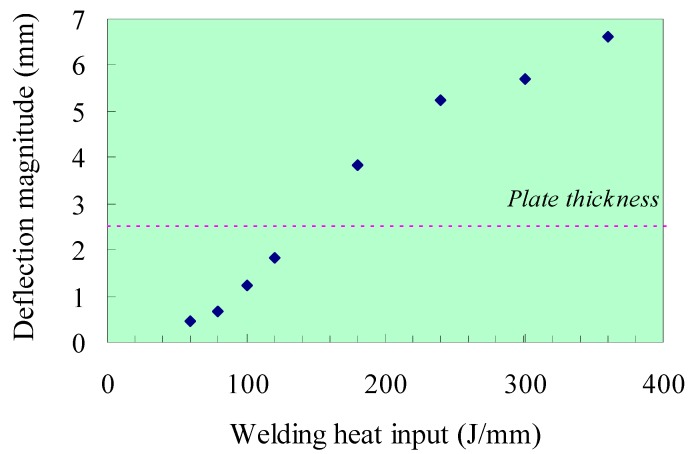
Relationship between welding heat input and maximum deflection.

**Figure 16:**
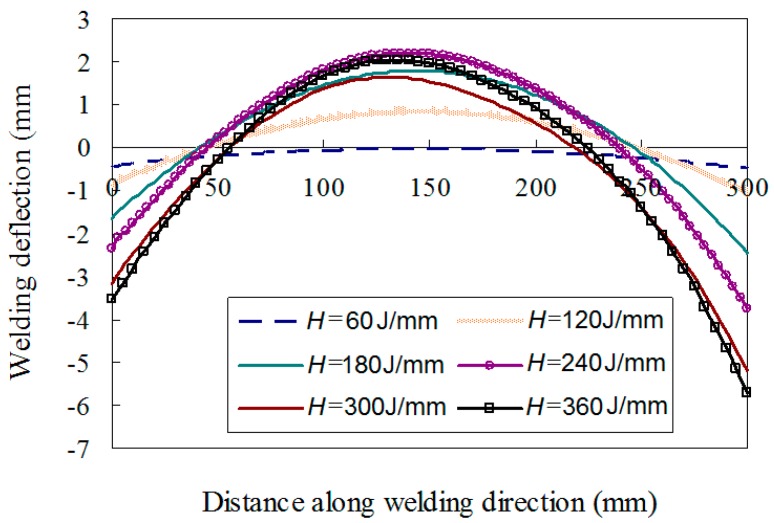
Deflection profile along the weld line.

**Figure 17:**
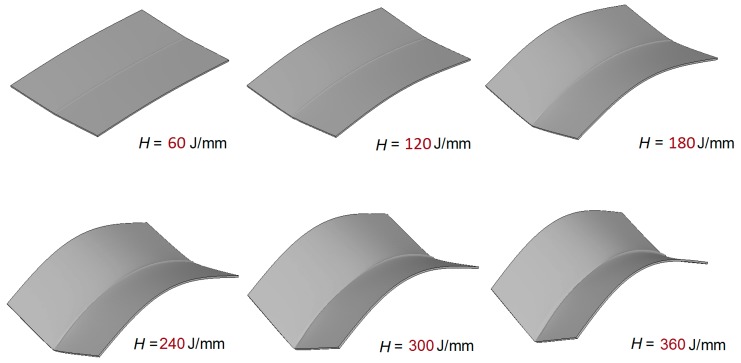
Welding distortion under various heat input condition (scale factor is 10).

Figure 15 shows the maximum deflection after welding is completed compared with the plate thickness. It can be concluded that the magnitude of the welding distortion greatly increases with the heat input when *H* is larger than 80 J/mm. The deflection of the weld line is shown in Figure 16 which indicates the single-wave mode distortion in all cases. Because the plate was welded under free conditions, the welding distortion behavior of plate is similar to that of a column under the axial load. Figure 17 shows the overall welding distortion of the plate with the scale factor of 10. From the figures, it can be confirmed that all distorted plates had a saddle shape. Obviously, the case with minimum welding heat input of 60 J/mm has an unnoticeable deformation while the experimental case gives a quite large distortion. In practical welding, the heat input should be minimized to avoid excessive out-of-plane distortion.

## 5. Conclusions

Based on the results by the numerical method and experimental measurement, the following conclusions can be drawn:

(1) The developed 3D thermal–mechanical FEM method can accurately predict the transient distortion in welding by considering the measured geometrical imperfection.
(2) The initial deformation of the plate has a large influence on both the deformation mode and the magnitude. The out-of-plane deformation in the opposite direction may be produced if the initial deformation is reversed.
(3) The distance between the welding torch and weldment tends to decrease if the initial shape of the plate is convex, which may interrupt the welding process if contact between electrode and plate occurs. To maintain the weld quality, adaptive arc length control or additional constraint should be introduced.
(4) The relationship between heat input and maximum deflection is not linear, there is a critical heat input beyond which the out-of-plane distortion starts to grow very quickly. Heat input can be optimized with the aid of numerical simulations to avoid the excessive welding distortion.

## Figures and Tables

**Table 1:** Welding parameters.

Welding	Current (I)	Voltage (U)	Welding Speed (*v*)
TIG	130 A	16 V	30 cm/min
